# Supplementary material for: Adverse childhood experiences, adult depression, and suicidal ideation in rural Uganda: A cross-sectional, population-based study
Source: PLoS Med. 2021 May 12;18(5):e1003642. doi: 10.1371/journal.pmed.1003642 (PMC8153443; doi:10.1371/journal.pmed.1003642)
Supplement: S1 Text — (DOCX) [file pmed.1003642.s002.docx]

**S1 Text.** Modified Adverse Childhood Experiences – International Questionnaire (ACE-IQ).

Now I would like to ask you about different things that may have happened to you while you were growing up, during your first 18 years of life. I know that this can be a sensitive issue. Please remember, as we discussed with you when you first agreed to participate in this study, all of your answers will be kept completely confidential and will not be told to anyone. If we should come to any question that you don’t want to answer, just let me know and we will skip to the next question. Also remember that I am not going to ask you the names of any people; for research purposes we only want to know whether or not these things happened to you during your first 18 years of life.

1. During the first 18 years of your life, how often did a parent or other adult in the household verbally abuse you, put you down, or humiliate you? (Never / Once / A few times / Frequently)
2. During the first 18 years of your life, how often did a parent or other adult in the household act in a way that made you afraid that he or she might physically harm you? (Never / Once / A few times / Frequently)
3. During the first 18 years of your life, how often did a parent or other adult in the household push, grab, or slap you, or throw objects at you to hurt you? (Never / Once / A few times / Frequently)
4. During the first 18 years of your life, how often did a parent or other adult in the household hit you so hard that you had marks or were injured? (Never / Once / A few times / Frequently)
5. During the first 18 years of your life, how often did a parent or person at least 5 years older than you touch your body in a sexual way, or make you touch their body in a sexual way? (Never / Once / A few times / Frequently)
6. During the first 18 years of your life, how often did a parent or person at least 5 years older than you have sex with you or try to have sex with you? (Never / Once / A few times / Frequently)
7. During the first 18 years of your life, were your parents ever separated or divorced? (Yes / No)
8. During the first 18 years of your life, how often did you observe another adult in the household push, grab, or slap your mother/grandmother/other female guardian or throw objects at her to hurt her? (Never / Once / A few times / Frequently)
9. During the first 18 years of your life, how often did you observe another adult in the household kick, bite, or punch your mother/grandmother/other female guardian? (Never / Once / A few times / Frequently)
10. During the first 18 years of your life, how often did you observe another adult in the household threaten your mother or grandmother with a knife, machete, or other weapon? (Never / Once / A few times / Frequently)
11. For how much of the first 18 years of your life did you live with an adult in the household who was a problem drinker or an alcoholic, or with an adult who used drugs? (Never / Part / All)
12. For how much of the first 18 years of your life did you live with an adult in the household who was depressed or mentally ill, or with an adult who had attempted suicide? (Never / Part / All)
13. During the first 18 years of your life, did a parent or other adult in the household ever get sent to prison or jail? (Yes / No)
14. During the first 18 years of your life, how often did you go a whole day without eating anything because there was not enough food in the household? (Never / Once / A few times / Frequently)
15. During the first 18 years of your life, how often did you go to bed hungry because there was not enough food in the household? (Never / Once / A few times / Frequently)
16. During the first 18 years of your life, how often did you go to bed thirsty because there was not enough water in the household? (Never / Once / A few times / Frequently)
