# Supplementary material for: Adverse childhood experiences, adult depression, and suicidal ideation in rural Uganda: A cross-sectional, population-based study
Source: PLoS Med. 2021 May 12;18(5):e1003642. doi: 10.1371/journal.pmed.1003642 (PMC8153443; doi:10.1371/journal.pmed.1003642)
Supplement: S2 Text — (DOCX) [file pmed.1003642.s003.docx]

**S2 Text.** Calculating the cumulative ACEs score from the modified version of the ACE-IQ – Binary Version.

If the participant responded that they ever experienced an event described in the corresponding questions for that category, mark a 1 in the response column. At the end of scoring, each of the 9 cells in the response column should be filled with a 0 or 1.

| **Category** | **Q** | **Written Question** | **Response** |
| --- | --- | --- | --- |
| *Physical Abuse* | Q3  Q4 | During the first 18 years of your life, did a parent or other adult in the household push, grab, or slap you, or throw objects at you to hurt you?  OR  During the first 18 years of your life, did a parent or other adult in the household hit you so hard that you had marks or were injured?  Yes No |  |
| *Verbal or Emotional Abuse* | Q1  Q2 | During the first 18 years of your life, did a parent or other adult in the household verbally abuse you, put you down, or humiliate you?  OR  During the first 18 years of your life, did a parent or other adult in the household act in a way that made you afraid that he or she might physically harm you?  Yes .No |  |
| *Attempted or Enacted Sexual Abuse* | Q5  Q6 | During the first 18 years of your life, did a parent or person at least 5 years older than you touch your body in a sexual way, or make you touch their body in a sexual way?  OR  During the first 18 years of your life, did a parent or person at least 5 years older than you have sex with you or try to have sex with you?  Yes No |  |
| *Residence with an Adult Who Used Alcohol or Drugs* | Q11 | For the first 18 years of your life did you live with an adult in the household who was a problem drinker or an alcoholic, or with an adult who used drugs?  Yes No |  |
| *Residence with an Adult Who Had Mental Illness or Who Attempted Suicide* | Q12 | For the first 18 years of your life did you live with an adult in the household who was depressed or mentally ill, or with an adult who had attempted suicide?  Yes No |  |
| *Parents Separated or Divorced* | Q7 | During the first 18 years of your life, were your parents ever separated or divorced?  Yes No |  |
| *Residence with an Adult Who was Sent to Prison or Jail* | Q13 | During the first 18 years of your life, did a parent or other adult in the household ever get sent to prison or jail?  Yes No |  |
| *Observed Violence toward Mother or Grandmother* | Q8  Q9  Q10 | During the first 18 years of your life, did you observe another adult in the household push, grab, or slap your mother/grandmother/other female guardian or throw objects at her to hurt her?  OR  During the first 18 years of your life, did you observe another adult in the household kick, bite, or punch your mother/grandmother/other female guardian?  OR  During the first 18 years of your life, did you observe another adult in the household threaten your mother or grandmother with a knife, machete, or other weapon?  Yes No |  |
| *Food and/or Water Insecurity* | Q14  Q15  Q16 | During the first 18 years of your life, did you go a whole day without eating anything because there was not enough food in the household?  OR  During the first 18 years of your life, did you go to bed hungry because there was not enough food in the household?  OR  During the first 18 years of your life, did you go to bed thirsty because there was not enough water in the household?  Yes No |  |
|  |  | **Total:** |  |
