# Supplementary material for: Adverse childhood experiences, adult depression, and suicidal ideation in rural Uganda: A cross-sectional, population-based study
Source: PLoS Med. 2021 May 12;18(5):e1003642. doi: 10.1371/journal.pmed.1003642 (PMC8153443; doi:10.1371/journal.pmed.1003642)
Supplement: S3 Text — (DOCX) [file pmed.1003642.s004.docx]

**S3 Text.** Methods

*Prespecified Analysis Plan*

No prospective protocol was published or registered for this observational study. However, we followed a clear analysis plan. The inclusion criteria for the study were established at the outset, in that a decision was made to include all eligible adults in the analysis, with no exclusions. The outcomes were prespecified at the outset. The statistical analyses were determined at the outset and were not changed. The various continuous and categorical specifications of the explanatory variables were prespecified, with minor changes made during peer review.

ACEs were assessed using a modified version of the ACE-IQ, and depression symptom severity and suicidal ideation were measured using the HSCL-D. We applied a previously developed algorithm to identify major depressive disorder based on the DSM-5. Additionally, participants reported their age, sex, highest level of educational attainment, marital status, HIV status, and household asset wealth.

To estimate bivariate associations between the cumulative number of ACEs and depression symptom severity based on the HSCL-D, we fitted a linear regression model with the cumulative number of ACEs as the explanatory variable. We re-fitted the model adjusting for the covariates listed above. To determine if associations held up across the age range, we first fitted a multivariable linear regression model containing a product term between the cumulative number of ACEs and age, specified as a continuous variable. We then conducted analyses stratified by age bin (younger adults, adults, older adults). We conducted similar analyses (Poisson regression models with cluster-correlated robust estimates of variance) to estimate unadjusted and adjusted associations between the cumulative number of ACEs and major depressive disorder and suicidal ideation.

In secondary analyses, we grouped the cumulative ACEs score into four categories and fitted the same linear and Poisson regression models. We then estimated associations between each type of ACE and depression symptom severity, major depressive disorder, and suicidal ideation. We probed the robustness of our findings to confounding by unobserved variables by calculating the e-value, or the minimum strength of association on the risk ratio scale that would be needed for an unobserved confounder to have with both the exposure and the outcome, conditional on the covariates, to explain away the observed associations. All analyses described above were adjusted for clustering at the village level. Changes that resulted from the peer review process are outlined below.

During the peer review process, we revised the scoring method for the modified ACE-IQ. On the initial submission, we had scored the ACE-IQ such that each of the 16 items contributed to the total score, which ranged from 0 to 16. Following reviewer feedback, and to more closely approximate the scoring of the original ACE-IQ, we grouped the items into nine types of ACEs: 1) physical abuse; 2) verbal abuse; 3) attempted or enacted sexual abuse; 4) residence with an adult who used alcohol or drugs; 5) residence with an adult who had mental illness or who attempted suicide; 6) parents separated or divorced; 7) residence with an adult who was sent to prison or jail; 8) observed violence toward mother or grandmother; and 9) food and/or water insecurity. Each of the nine types of ACEs contributed to the revised cumulative score, which ranged from 0 to 9. If, for example, a participant reported either *or* both of the two physical abuse experiences, s/he received a score of 1 for physical abuse.

This change to the scoring also affected the scoring for the categorical ACE score. Based on the updated median number and interquartile range of ACEs, we specified new category cutoffs: 0-1, 2-3, 4-5, and ≥6 ACEs. Finally, in the linear and Poisson regression models between each individual ACE and depression symptom severity, major depressive disorder, and suicidal ideation, we fitted models for each of the 9 types of ACEs rather than each of the 16 individual items.

As a result of these scoring changes, the estimated regression coefficients derived from the primary linear and Poisson regression models (Table 3) changed little, and the substantive conclusions of our analysis remained qualitatively similar. However, in the regression models estimating the associations between the ACEs categories and suicidal ideation, we no longer found statistically significant associations between the highest category of ACE (≥6 ACEs) and suicidal ideation. In the regression models estimating the associations between each of the nine types of ACEs and depression and suicidality, we estimated statistically significant associations between every type of ACE and major depressive disorder (9 of 9 models compared with 15 of 16 models in the analyses on the initial submission) and between two ACEs and suicidal ideation (2 of 9 models rather than 1 of 16 models in the analyses on the initial submission).

In response to reviewer feedback, we also added a sensitivity analysis during the peer review process. We replicated the primary analyses (associations between cumulative ACEs score and depression symptom severity, major depressive disorder, and suicidal ideation), adjusting for clustering at the household level rather than at the village level. The estimates remained qualitatively similar when we clustered the standard errors at the household level.
