# Supplementary material for: Adverse childhood experiences, adult depression, and suicidal ideation in rural Uganda: A cross-sectional, population-based study
Source: PLoS Med. 2021 May 12;18(5):e1003642. doi: 10.1371/journal.pmed.1003642 (PMC8153443; doi:10.1371/journal.pmed.1003642)
Supplement: S1 Table — (DOCX) [file pmed.1003642.s005.docx]

**S1 Table.** Unadjusted linear and Poisson regression models estimating associations between cumulative number of ACEs and depression symptom severity, major depressive disorder, and suicidal ideation.

|  | **Depression Symptom Severity** | | **Major Depressive Disorder** | | **Suicidal Ideation** | |
| --- | --- | --- | --- | --- | --- | --- |
|  | **b**  **(95% CI)** | ***p*-value** | **RR**  **(95% CI)** | ***p*-value** | **RR**  **(95% CI)** | ***p*-value** |
| **Cumulative No. ACEs** | 0.046  (0.038-0.053) | <0.001 | 1.184  (1.102-1.271) | <0.001 | 1.164  (1.008-1.343) | 0.038 |
| **Constant** | 1.329  (1.281-1.377) | <0.001 | 0.052  (0.034-0.079) | <0.001 | 0.010  (0.005-0.018) | <0.001 |
| **Observations** | 1,626 | | 1,626 | | 1,626 | |
| **R^2^ and Pseudo R^2^** | 0.058 | | 0.022 | | 0.012 | |
| Abbreviations: b, beta coefficient; RR, relative risk; CI, confidence interval; ACEs, adverse childhood experiences | | | | | | |
