# Supplementary material for: Adverse childhood experiences, adult depression, and suicidal ideation in rural Uganda: A cross-sectional, population-based study
Source: PLoS Med. 2021 May 12;18(5):e1003642. doi: 10.1371/journal.pmed.1003642 (PMC8153443; doi:10.1371/journal.pmed.1003642)
Supplement: S2 Table — (DOCX) [file pmed.1003642.s006.docx]

**S2 Table.** Adjusted linear and Poisson regression models estimating associations between number of ACEs and depression symptom severity, major depressive disorder, and suicidal ideation, using standard errors clustered at the household level.

|  | **Depression Symptom Severity** | | **Major Depressive Disorder** | | **Suicidal Ideation** | |
| --- | --- | --- | --- | --- | --- | --- |
|  | **Adjusted b (95% CI)** | ***p*-value** | **Adjusted RR (95% CI)** | ***p*-value** | **Adjusted RR (95% CI)** | ***p*-value** |
| **Cumulative No. ACEs** | 0.050 (0.041-0.059) | <0.001 | 1.190 (1.114-1.270) | <0.001 | 1.146 (0.963-1.364) | 0.126 |
| **Female** | 0.178 (0.141-0.215) | <0.001 | 1.858 (1.338-2.580) | <0.001 | 2.620 (1.065-6.444) | 0.036 |
| **Age (years)** | 0.003 (0.001-0.004) | <0.001 | 1.002 (0.994-1.011) | 0.586 | 0.989 (0.963-1.015) | 0.395 |
| **Completed Primary School** | -0.087 (-0.137-0.036) | 0.001 | 0.471 (0.333-0.667) | <0.001 | 0.365 (0.149-0.896) | 0.028 |
| **Married** | -0.068 (-0.109- -0.028) | 0.001 | 0.712 (0.528-0.960) | 0.026 | 1.178 (0.536-2.592) | 0.683 |
| **HIV Positive** | -0.035 (-0.098-0.027) | 0.268 | 0.809 (0.520-1.261) | 0.350 | 1.071 (0.380-3.021) | 0.896 |
| **Wealth Quintile Category** |  |  |  |  |  |  |
| Poorest |  |  |  |  |  |  |
| 2nd | -0.060 (-0.124-0.004) | 0.066 | 0.783 (0.517-1.186) | 0.248 | 0.311 (0.086-1.130) | 0.076 |
| 3rd | -0.040 (-0.104-0.023) | 0.215 | 0.804 (0.518-1.246) | 0.329 | 0.856 (0.328-2.238) | 0.752 |
| 4th | -0.068 (-0.132- -0.003) | 0.040 | 0.644 (0.392-1.059) | 0.083 | 0.284 (0.065-1.242) | 0.094 |
| Richest | -0.035 (-0.105-0.035) | 0.330 | 1.008 (0.630-1.614) | 0.972 | 1.019 (0.347-2.991) | 0.973 |
| **Constant** | 1.246 (1.161-1.330) | <0.001 | 0.065 (0.034-0.122) | <0.001 | 0.018 (0.004-0.090) | <0.001 |
| **Observations** | 1,602 | | 1,602 | | 1,602 | |
| **R^2^ and Pseudo R^2^** | 0.157 | | 0.076 | | 0.080 | |
| Abbreviations: b, beta coefficient; RR, relative risk; CI, confidence interval; ACEs, adverse childhood experiences | | | | | | |
