# Supplementary material for: Adverse childhood experiences, adult depression, and suicidal ideation in rural Uganda: A cross-sectional, population-based study
Source: PLoS Med. 2021 May 12;18(5):e1003642. doi: 10.1371/journal.pmed.1003642 (PMC8153443; doi:10.1371/journal.pmed.1003642)
Supplement: S3 Table — (DOCX) [file pmed.1003642.s007.docx]

**S3 Table.** Linear regression model with product term between cumulative number of ACEs and age (specified as a continuous variable), and linear regression models estimating associations between cumulative number of ACEs and depression symptom severity, stratified by age category.

|  | **Depression Symptom Severity** | | | | | | | | | | | | | |
| --- | --- | --- | --- | --- | --- | --- | --- | --- | --- | --- | --- | --- | --- | --- |
|  | **Product Term –**  **ACEs by Age** | | **Younger Adults**  **(17-26 years)** | | | | **Adults**  **(27-39 years)** | | | | **Older Adults**  **(40+ years)** | | | |
|  | **Adjusted b**  **(95% CI)** | ***p*-value** | **b**  **(95% CI)** | ***p*-value** | **Adjusted b**  **(95% CI)** | ***p*-value** | **b**  **(95% CI)** | ***p*-value** | **Adjusted b**  **(95% CI)** | ***p*-value** | **b**  **(95% CI)** | ***p*-value** | **Adjusted b**  **(95% CI)** | ***p*-value** |
| **Cumulative No. ACEs** | 0.091  (0.074-0.109) | <0.001 | 0.059  (0.045-0.072) | <0.001 | 0.060  (0.047-0.073) | <0.001 | 0.068  (0.059-0.076) | <0.001 | 0.064  (0.056-0.071) | <0.001 | 0.030  (0.019-0.041) | <0.001 | 0.031  (0.017-0.046) | 0.001 |
| **Female** | 0.180  (0.136-0.223) | <0.001 |  |  | 0.177  (0.073-0.280) | 0.005 |  |  | 0.186  (0.094-0.279) | 0.002 |  |  | 0.146  (0.076-0.215) | 0.002 |
| **Age (years)** | 0.006  (0.005-0.007) | <0.001 |  |  | 0.019  (0.003-0.035) | 0.026 |  |  | -0.005  (-0.022-0.012) | 0.484 |  |  | 0.001  (-0.002-0.004) | 0.437 |
| **Completed Primary School** | -0.084  (-0.168- -0.000) | 0.050 |  |  | -0.008  (-0.097-0.081) | 0.834 |  |  | -0.115  -0.229- -0.001) | 0.048 |  |  | -0.099  (-0.211-0.012) | 0.072 |
| **Married** | -0.070  (-0.116- -0.025) | 0.008 |  |  | -0.069  (-0.179-0.041) | 0.180 |  |  | -0.051  (-0.143-0.042) | 0.236 |  |  | -0.144  (-0.256- -0.031) | 0.019 |
| **HIV Positive** | -0.034  (-0.097-0.029) | 0.243 |  |  | -0.152  (-0.335-0.032) | 0.091 |  |  | 0.128  (-0.045-0.301) | 0.123 |  |  | -0.119  (-0.214- -0.025) | 0.020 |
| **Wealth Quintile Category** |  |  |  |  |  |  |  |  |  |  |  |  |  |  |
| Poorest |  |  |  |  |  |  |  |  |  |  |  |  |  |  |
| 2nd | -0.063  (-0.127-0.001) | 0.053 |  |  | -0.079  (-0.198-0.040) | 0.161 |  |  | -0.070  (-0.114- -0.025) | 0.008 |  |  | -0.043  (-0.199-0.112) | 0.531 |
| 3rd | -0.042  (-0.105-0.020) | 0.153 |  |  | -0.075  (-0.183-0.033) | 0.144 |  |  | 0.004  (-0.125-0.132) | 0.950 |  |  | -0.043  (-0.145-0.060) | 0.357 |
| 4th | -0.072  (-0.131- -0.014) | 0.022 |  |  | -0.097  (-0.260-0.066) | 0.203 |  |  | -0.039  (-0.170-0.093) | 0.508 |  |  | -0.055  (-0.185-0.075) | 0.350 |
| Richest | -0.032  (-0.120-0.055) | 0.411 |  |  | -0.092  (-0.283-0.099) | 0.001 |  |  | 0.008  (-0.119-0.136) | 0.880 |  |  | 0.008  (-0.137-0.153) | 0.905 |
| Cumulative No. ACEs by Age | -0.001  (-0.0014- -0.0006) | <0.001 |  |  |  | |  |  |  |  |  |  |  | |
| **Constant** | 1.115  (1.039-1.190) | <0.001 | 1.228  (1.147-1.309) | <0.001 | 0.806  (0.430-1.183) | 0.001 | 1.222  (1.165-1.279) | <0.001 | 1.429  (0.854-2.003) | 0.001 | 1.421  (1.351-1.491) | <0.001 | 1.458  (1.168-1.747) | <0.001 |
| **Observations** | 1,602 | | 409 | | 409 | | 488 | | 488 | | 705 | | 705 | |
| **R^2^** | 0.165 | | 0.104 | | 0.163 | | 0.123 | | 0.214 | | 0.024 | | 0.148 | |
| Abbreviations: b, beta coefficient; CI, confidence interval; ACEs, adverse childhood experiences  The adjusted models are each adjusted for sex, age, education, marital status, HIV status, and household asset wealth quintile category. | | | | | | | | | | | | | | |
