# Supplementary material for: Adverse childhood experiences, adult depression, and suicidal ideation in rural Uganda: A cross-sectional, population-based study
Source: PLoS Med. 2021 May 12;18(5):e1003642. doi: 10.1371/journal.pmed.1003642 (PMC8153443; doi:10.1371/journal.pmed.1003642)
Supplement: S4 Table — (DOCX) [file pmed.1003642.s008.docx]

**S4 Table.** Unadjusted linear and Poisson regression models estimating associations between ACEs category and depression symptom severity, major depressive disorder, and suicidal ideation.

|  | **Depression**  **Symptom Severity** | | **Major Depressive Disorder** | | **Suicidal Ideation** | |
| --- | --- | --- | --- | --- | --- | --- |
|  | **b**  **(95% CI)** | ***p*-value** | **RR**  **(95% CI)** | ***p*-value** | **RR**  **(95% CI)** | ***p*-value** |
| **ACEs Category** |  |  |  |  |  |  |
| Lowest (0-1 ACE) |  |  |  |  |  |  |
| Low (2-3 ACEs) | 0.077  (0.009-0.145) | 0.031 | 1.331  (0.886-2.000) | 0.168 | 1.935  (0.759-4.932) | 0.167 |
| High (4-5 ACEs) | 0.116  (0.069-0.163) | 0.001 | 1.448  (1.030-2.036) | 0.033 | 1.293  (0.443-3.769) | 0.638 |
| Highest (≥6 ACEs) | 0.287  (0.227-0.346) | <0.001 | 2.616  (1.725-3.967) | <0.001 | 2.669  (0.973-7.320) | 0.057 |
| **Constant** | 1.375  (1.324-1.425) | <0.001 | 0.065  (0.042-0.099) | <0.001 | 0.010  (0.005-0.021) | <0.001 |
| **Observations** | 1,626 | | 1,626 | | 1,626 | |
| **R^2^ and Pseudo R^2^** | 0.050 | | 0.017 | | 0.012 | |
| Abbreviations: b, beta coefficient; RR, relative risk; CI, confidence interval; ACEs, adverse childhood experiences | | | | | | |
