# Supplementary material for: Adverse childhood experiences, adult depression, and suicidal ideation in rural Uganda: A cross-sectional, population-based study
Source: PLoS Med. 2021 May 12;18(5):e1003642. doi: 10.1371/journal.pmed.1003642 (PMC8153443; doi:10.1371/journal.pmed.1003642)
Supplement: S5 Table — (DOCX) [file pmed.1003642.s009.docx]

**S5 Table.** Linear and Poisson regression models estimating associations between each type of ACE and depression symptom severity, major depressive disorder, and suicidal ideation.

|  | **HSCL-D Depression Symptom Severity** | | | | **Major Depressive Disorder** | | | | **Suicidal Ideation** | | | |
| --- | --- | --- | --- | --- | --- | --- | --- | --- | --- | --- | --- | --- |
|  | **b**  **(95% CI)** | ***p-*value** | **Adjusted b**  **(95% CI)** | ***p*-value** | **RR**  **(95% CI)** | ***p*-value** | **Adjusted RR**  **(95% CI)** | ***p*-value** | **RR**  **(95% CI)** | ***p*-value** | **Adjusted RR**  **(95% CI)** | ***p*-value** |
| **Physical Abuse** | 0.078  (0.038-0.119) | 0.003 | 0.095  (0.040-0.150) | 0.005 | 1.408  (1.088-1.821) | 0.009 | 1.529  (1.178-1.984) | 0.001 | 1.504  (0.925-2.446) | 0.100 | 1.485  (0.930-2.371) | 0.097 |
|  |  |  |  |  |  |  |  |  |  |  |  |  |
| **Verbal or Emotional Abuse** | 0.088  (0.050-0.127) | 0.001 | 0.116  (0.063-0.170) | 0.001 | 1.509  (1.150-1.979) | 0.003 | 1.685  (1.236-2.297) | 0.001 | 0.652  (0.292-1.459) | 0.298 | 0.661  (0.286-1.526) | 0.332 |
|  |  |  |  |  |  |  |  |  |  |  |  |  |
| **Attempted or Enacted Sexual Abuse** | 0.206  (0.132-0.279) | <0.001 | 0.193  (0.127-0.259) | <0.001 | 1.569  (1.125-2.188) | 0.008 | 1.487  (1.102-2.005) | 0.009 | 1.698  (1.006-2.868) | 0.048 | 1.573  (0.898-2.754) | 0.113 |
|  |  |  |  |  |  |  |  |  |  |  |  |  |
| **Residence with an Adult Who Used Alcohol or Drugs** | 0.086  (0.032-0.140) | 0.007 | 0.088  (0.033-0.142) | 0.007 | 1.422  (1.072-1.886) | 0.014 | 1.451  (1.074-1.962) | 0.015 | 1.354  (0.606-3.025) | 0.460 | 1.306  (0.606-2.814) | 0.496 |
|  |  |  |  |  |  |  |  |  |  |  |  |  |
| **Residence with an Adult Who Had Mental Illness or Who Attempted Suicide** | 0.136  (0.087-0.185) | <0.001 | 0.137  (0.074-0.200) | 0.001 | 1.579  (1.253-1.989) | <0.001 | 1.576  (1.243-2.000) | <0.001 | 1.414  (0.760-2.631) | 0.274 | 1.361  (0.734-2.524) | 0.328 |
|  |  |  |  |  |  |  |  |  |  |  |  |  |
| **Parents Separated or Divorced** | 0.139  (0.092-0.185) | <0.001 | 0.134  (0.079-0.189) | 0.001 | 1.584  (1.194-2.101) | 0.001 | 1.500  (1.114-2.021) | 0.008 | 1.664  (0.828-3.342) | 0.152 | 1.414  (0.675-2.963) | 0.359 |
|  |  |  |  |  |  |  |  |  |  |  |  |  |
| **Residence with an Adult Who was Sent to Prison or Jail** | 0.070  (0.018-0.122) | 0.015 | 0.091  (0.050-0.131) | 0.001 | 1.300  (0.906-1.864) | 0.154 | 1.457  (1.083-1.961) | 0.013 | 2.554  (1.608-4.055) | <0.001 | 2.654  (1.646-4.278) | <0.001 |
|  |  |  |  |  |  |  |  |  |  |  |  |  |
| **Observed Violence Toward Mother or Grandmother** | 0.139  (0.099-0.179) | <0.001 | 0.131  (0.088-0.174) | <0.001 | 1.598  (1.205-2.120) | 0.001 | 1.473  (1.104-1.964) | 0.008 | 1.188  (0.429-3.293) | 0.740 | 1.090  (0.420-2.828) | 0.859 |
|  |  |  |  |  |  |  |  |  |  |  |  |  |
| **Food and/or Water Insecurity** | 0.159  (0.108-0.211) | <0.001 | 0.187  (0.137-0.238) | <0.001 | 1.958  (1.576-2.433) | <0.001 | 2.089  (1.657-2.633) | <0.001 | 2.034  (1.097-3.769) | 0.024 | 1.882  (1.155-3.065) | 0.011 |
|  |  |  |  |  |  |  |  |  |  |  |  |  |
| Abbreviations: b, beta coefficient; RR, relative risk; CI, confidence interval; HSCL-D, Hopkins Symptom Checklist for Depression | | | | | | | | | | | | |
| Each row/column cell provides the estimate of the association between the adversity listed in the row header and the outcome listed in the column header, and therefore represents the output of a separate regression model. In the adjusted analyses columns, the multivariable regression models were each adjusted for sex, age, education, marital status, HIV status and household asset wealth quintile category. Thus, this table provides output from 54 regression models in total (27 unadjusted and 27 adjusted). | | | | | | | | | | | | |
