# Supplementary material for: Adverse childhood experiences, adult depression, and suicidal ideation in rural Uganda: A cross-sectional, population-based study
Source: PLoS Med. 2021 May 12;18(5):e1003642. doi: 10.1371/journal.pmed.1003642 (PMC8153443; doi:10.1371/journal.pmed.1003642)
Supplement: Attachment — Submitted filename: satinsky.pdf [file pmed.1003642.s010.pdf]

This paper describes the association between reported childhood adversity and adult depression in rural Uganda.

I was asked for a statistical report and I interpret that to include all aspects of the design and conduct of the study.

Points of detail

Page 5 I am not sure what an emancipated minor is. Presumably not freed from slavery. Does it mean living without a parent or legal guardian?

Page 6 It seems surprising not to ask about abuse outside the household. Large–scale abuse of children by clerics and sporting coaches has been revealed in the media both in my country and that of the authors and elsewhere. Perhaps I am unduly cynical but is it really just a phenomenon of rich countries?

Page 6 The women do not seem to have been asked if they were cut. Perhaps it is either so common or so rare in Uganda that associations would not have been revealed.

Page 7 I think the authors probably used five categories rather than the four quintiles although that would have been possible. This leads to possible ambiguity. If fifths is not acceptable quintile categories is also used.

Page 8 A preliminary test for interaction would seem indicated before stratifying by age group. But why stratify? Categorising an essentially continuous variable wastes information (Altman and Royston, 2006; Royston et al., 2006) and leads to models which are often implausible as they predict the effect remaining flat within categories and then jumping to a new value at the category boundary.

Page 12, Table 2 There are nine categories here not the 16 mentioned on page 6. I think I may be able to guess what is going on especially after looking at S2 but perhaps the authors might make it explicit here. If I am wrong and some have been dropped then that would seem sub–optimal.

Page 13 I would not have said that the difference between 0.042, 0.043 and 0.022 were minor as the last is about half the other two.

Page 18 The prevalences here do not seem very close to those in Table 2. The authors do mention as a possible limitation not being able to

ensure confidentiality of the interviews but did reduce the prevalence of all the ACE relative to epidemiological studies?

Point of more substance

In pages 19 and 20 the authors present their recommendations for the health service in Uganda. It seems to me that their research design has not justified this as it has not ruled out reverse causation. Presumably many of the people currently depressed may have had mental health problems before age 18 although not necessarily depression. That may have influenced the way people behaved towards them leading to at least some of the items on the ACE schedule becoming more prevalent or it may be that their childhood problems modified their perception of the world and events in it. In either of those cases the recommendation would presumably be to increase funding for child and adolescent mental health services rather than reducing adverse events.

Summary

No major issues.

Michael Dewey

References

D G Altman and P Royston. The cost of dichotomising continuous variables. *British Medical Journal*, 332:1080, 2006.

P Royston, D G Altman, and W Sauerbrei. Dichotomizing continuous predictors in multiple regression: a bad idea. *Statistics in Medicine*, 25:127–141, 2006.
