# Supplementary material for: Adverse childhood experiences, adult depression, and suicidal ideation in rural Uganda: A cross-sectional, population-based study
Source: PLoS Med. 2021 May 12;18(5):e1003642. doi: 10.1371/journal.pmed.1003642 (PMC8153443; doi:10.1371/journal.pmed.1003642)
Supplement: Attachment — Submitted filename: acedepression_response-to-reviewers_plosmed_20201211.docx [file pmed.1003642.s011.docx]

**Response to Reviewers**

**Requests from the editors:**

**1. Data availability statement: Thank you for your willingness to make your data and code available. At this time, please provide information on where the anonymized data may be accessed.**

RESPONSE: We will be making the data available on GitHub, and we will provide a URL once the manuscript is accepted for publication.

**2. Protocol: Did your study have a prospective protocol or analysis plan? Please state this (either way) early in the Methods section.**

RESPONSE: We did not have a prospective protocol. At the beginning of the Data Analysis section of the Methods, we state that while we did not have a registered study protocol, we followed a clear analysis plan.

“The analysis was not pre-registered, but we followed a prespecified analysis plan and tracked any deviations that resulted from peer review (S3 Appendix).” (page 13)

**a) If a prospective analysis plan (from your funding proposal, IRB or other ethics committee submission, study protocol, or other planning document written before analyzing the data) was used in designing the study, please include the relevant prospectively written document with your revised manuscript as a Supporting Information file to be published alongside your study, and cite it in the Methods section. A legend for this file should be included at the end of your manuscript.**

RESPONSE: At the beginning of the Data Analysis section of the Methods, we provide a reference to our analysis plan (S3 Appendix). This file is cited as a legend at the end of the manuscript.

**b) If no such document exists, please make sure that the Methods section transparently describes when analyses were planned, and when/why any data-driven changes to analyses took place.**

RESPONSE: We have included the data analysis plan (Materials and Methods; S3 Appendix) as a Supporting Information file.

**c) In either case, changes in the analysis-- including those made in response to peer review comments-- should be identified as such in the Methods section of the paper, with rationale.**

RESPONSE: In the Appendix, we not that we updated the scoring criteria for the modified version of the ACE-IQ in response to reviewer feedback.

“During the peer review process, we revised the scoring method for the modified ACE-IQ. On the initial submission, we had scored the ACE-IQ such that each of the 16 items contributed to the total score, which ranged from 0 to 16. Following reviewer feedback, and to more closely approximate the scoring of the original ACE-IQ, we grouped the items into nine types of ACE: 1) physical abuse; 2) verbal abuse; 3) attempted or enacted sexual abuse; 4) residence with an adult who used alcohol or drugs; 5) residence with an adult who had mental illness or who attempted suicide; 6) parents separated or divorced; 7) residence with an adult who was sent to prison or jail; 8) observed violence toward mother or grandmother; and 9) food and/or water insecurity. Each of the 9 types of ACEs contributed to the revised cumulative score, which ranged from 0 to 9. If, for example, a participant reported either or both of the two physical abuse experiences, s/he received a score of 1 for physical abuse.”

We also describe how this change in the analysis resulted in slight changes to our estimates:

“This change to the scoring also affected the scoring for the categorical ACE score. Based on the updated median number and interquartile range of ACEs, we specified new category cutoffs: 0-1, 2-3, 4-5, and ≥6 ACEs. Finally, in the linear and Poisson regression models between each individual ACE and depression symptom severity, major depressive disorder, and suicidal ideation, we fitted models for each of the 9 types of ACEs rather than each of the 16 individual items.

“As a result of these scoring changes, the estimated regression coefficients derived from the primary linear and Poisson regression models (Table 3) changed little, and the substantive conclusions of our analysis remained qualitatively similar. However, in the regression models estimating the associations between the ACE categories and suicidal ideation, we no longer found statistically significant associations between the highest category of ACE (≥6 ACEs) and suicidal ideation. In the regression models estimating the associations between each of the nine types of ACEs and depression and suicidality, we estimated statistically significant associations between every type of ACE and major depressive disorder (9 of 9 models compared with 15 of 16 models in the analyses on the initial submission) and between two ACEs and suicidal ideation (2 of 9 models rather than 1 of 16 models in the analyses on the initial submission).”

**3. Abstract: Methods and Findings: Early on in this section, please mention the key features of the study design (e.g. population and setting, number of participants, years during which the study took place, and main outcome measures). Please provide some summary demographic information pertaining to the study participants, including how outcome measures were obtained (ACEs numbers, depression symptom severity/MDD diagnosis, suicide ideation).**

RESPONSE: At the beginning of the Methods and Findings section of the Abstract, we now include additional information on the population, setting, number of participants, survey years, and main outcome measures.

“Between 2017 and 2018, research assistants visited the homes of 1,626 adult residents of Nyakabare Parish, a rural area in southwestern Uganda. ACEs were elicited using a modified version of the Adverse Childhood Experiences-International Questionnaire, and depression symptom severity and suicidal ideation were assessed using the Hopkins Symptom Checklist for Depression-15 (HSCLD-15). We applied a validated algorithm to determine major depressive disorder diagnoses. Overall, 1,458 participants (90%) had experienced at least one ACE, 159 participants (10%) met criteria for major depressive disorder, and 28 participants (1.7%) reported significant suicidal ideation.” (page 2)

**4. Abstract: Methods and Findings: For the reported results on number of ACEs and depressive symptoms, risk of major depressive disorder, and suicidal ideation, please provide the p values in addition to providing confidence intervals (please also define the abbreviation CI at first use).**

RESPONSE: Throughout the manuscript, we now include the p-values in addition to the 95% CIs for the adjusted associations between cumulative number of ACEs and depressive symptom severity, major depressive disorder, and suicidal ideation. We also define the abbreviation ‘CI’ at first use.

**5. Abstract: Methods and Findings: Please mention the important variables that are adjusted for in the analyses.**

RESPONSE: In the Methods and Findings section of the Abstract, we now list the variables adjusted for in the linear and Poisson regression models.

“In multivariable regression models adjusted for age, sex, primary school completion, marital status, self-reported HIV status, and household asset wealth, the cumulative number of ACEs was associated with greater depression symptom severity (b=0.050; 95% confidence interval [CI], 0.039-0.061; p<0.001), and increased risk for major depressive disorder (adjusted relative risk [ARR]=1.190; 95% CI, 1.109-1.276; p<0.001) and suicidal ideation (ARR=1.146; 95% CI, 1.001-1.311; p=0.048).” (page 2)

**6. Abstract: Methods and Findings: In the last sentence of the Abstract Methods and Findings section, please describe the main limitation(s) of the study's methodology.**

RESPONSE: In the last sentence of the Abstract Methods and Findings section, we now include a sentence with some of the key limitations.

“The limitations of the study include the reliance on retrospective self-report as well as the focus on ACEs that occurred within the household.” (page 3)

**7. Abstract: Conclusion: In the first sentence, we suggest you address the results of the study prior to mentioning the implications; the phrase "In this study, we observed ..." may be useful.**

RESPONSE: Thank you for this suggestion. We now begin the Conclusions section of the Abstract with the following sentence:

“In this whole-population, cross-sectional study of adults in rural Uganda, the cumulative number of ACEs had statistically significant associations with depression symptom severity, major depressive disorder, and suicidal ideation.” (page 3)

**8. Author Summary: At this stage, we ask that you include a short, non-technical Author Summary of your research to make findings accessible to a wide audience that includes both scientists and non-scientists. The Author Summary should immediately follow the Abstract in your revised manuscript. This text is subject to editorial change and should be distinct from the scientific abstract. Please see our author guidelines for more information:** [**https://secure-web.cisco.com/1tTcZonjx8qEtFxhVf5UnQ_IRh3n8J7HS5BYK2aeXodyXtSZPWD6ho9m5JS9oHSC_-bTJIlM4yZqxT8Qmy0LnJlT_p3gR3gu3ByKdzl_Hl5g6Z1AqSa_X20D7izWy5ItbtYYUNsXSFlBuXoCnzfzG_21rz7jZhKyMhbhrUfGXrAc1YM5asLonzziQ9OYOEc_EVYjrJStklfwI6uEyzNEKGTKybiLB-hgFsdQIBB8sQ829DuyMN2eqEtMAf5qjZ6JDkEZTO8aBsPG27zJNcbVTMA/https%3A%2F%2Fjournals.plos.org%2Fplosmedicine%2Fs%2Frevising-your-manuscript%23loc-author-summary**](https://secure-web.cisco.com/1tTcZonjx8qEtFxhVf5UnQ_IRh3n8J7HS5BYK2aeXodyXtSZPWD6ho9m5JS9oHSC_-bTJIlM4yZqxT8Qmy0LnJlT_p3gR3gu3ByKdzl_Hl5g6Z1AqSa_X20D7izWy5ItbtYYUNsXSFlBuXoCnzfzG_21rz7jZhKyMhbhrUfGXrAc1YM5asLonzziQ9OYOEc_EVYjrJStklfwI6uEyzNEKGTKybiLB-hgFsdQIBB8sQ829DuyMN2eqEtMAf5qjZ6JDkEZTO8aBsPG27zJNcbVTMA/https%3A%2F%2Fjournals.plos.org%2Fplosmedicine%2Fs%2Frevising-your-manuscript%23loc-author-summary)

RESPONSE: We now include a non-technical Author Summary on pages 4-5.

**“Why was this study done?**

- Depression is recognized globally as a leading cause of disability. Studies from high-income countries have shown robust associations between adverse childhood experiences (ACEs) and depression during adulthood.
- While studies from sub-Saharan Africa have demonstrated associations between ACEs and depression and suicidality among children, adolescents, and young adults, no study from this region has yet estimated the associations between ACEs and major depressive disorder and suicidal ideation within a whole-population sample of adults.

**What did the researchers do and find?**

- We conducted a cross-sectional, population-based study of 1,626 adults in rural Uganda, eliciting ACEs, current depression, and suicidal ideation through face-to-face interviews.
- The cumulative number of ACEs that occurred before age 18 had statistically significant associations with depression symptom severity, major depressive disorder, and suicidal ideation.
- Depression symptom severity and major depressive disorder had statistically significant associations with each of the nine types of ACEs. Suicidal ideation also had statistically significant associations with living with an adult who was sent to jail or prison during childhood and experiencing food and/or water insecurity during childhood.

**What do these findings mean?**

- Our interpretation of these findings raises implications for the development of policies and programs that support children, adolescents, and their families, and promote mental health.
- We are not able to determine the extent to which these associations are causal, and our analysis is susceptible to potential bias from the use of retrospective self-report of ACEs.”

**9. Throughout the text: Please use square brackets for in-text citations, like this [1].**

RESPONSE: All of our in-text citations are now reported with square brackets.

**10. Methods: Line 85: Please remove the trademark symbol.**

RESPONSE: The trademark symbol has been removed.

**11: Methods: Line 90: Please provide the modified version of the ACE-IQ and reference it along with your description as a supporting information file.**

RESPONSE: We have now included the modified version of the ACE-IQ, as well as the modified scoring guide, as supporting information files (S1 and S2 Appendix). As described in our response to Reviewer 3’s comment, we also include new information in the Methods section on our team’s process for modifying and scoring the ACE-IQ. This additional information is included on pages 10-11.

“We modified the ACE-IQ following an iterative process of focus group discussion with key informants. Some of the original ACE-IQ items were dropped because they were either poorly understood or thought to be less applicable for our population. Additional items on food and water insecurity were added because both food and water insecurity are common in this rural setting.” (pages 10-11)

“The 16 items on the ACE-IQ were then grouped into nine types of ACEs: 1) physical abuse; 2) verbal or emotional abuse; 3) attempted or enacted sexual abuse; 4) residence with an adult who used alcohol or drugs; 5) residence with an adult who had mental illness or who attempted suicide; 6) parents separated or divorced; 7) residence with an adult who was sent to prison or jail; 8) observed violence toward mother or grandmother; and 9) food and/or water insecurity. These nine binary variables were summed to calculate the cumulative number of ACEs (S2 Appendix).” (page 11)

**12. Methods: Line 119-123: As mentioned by a reviewer, please do provide some further description of the algorithm used to identify major depressive disorder, even though the references are provided.**

RESPONSE: We have added further description of the algorithm used to identify major depressive disorder. This information is provided on page 12.

“The original HSCL-D includes 15 items. Local modifications involved dropping one item (“feeling trapped”) and adding one item (“don’t care what happens to your health”). These 15 items were then categorized into the nine DSM ‘A’ criteria: A1) depressed mood, A2) diminished interest or pleasure, A3) significant weight loss or change in appetite, A4) insomnia or hypersomnia, A5) psychomotor agitation, A6) fatigue or loss of energy, A7) feeling worthless or guilty, A8) diminished ability to think or concentrate; and A9) recurrent thoughts of death. Participants who experienced an HSCL-D item ‘much’ or ‘very much’ in the past week were classified as meeting the respective DSM ‘A’ criteria. We then summed the total number of criteria met, out of a maximum of nine. If a participant reported at least 5 criteria, *and* met either the A1 criterion (depressed mood) *or* the A2 criterion (diminished interest or pleasure), s/he was identified as likely meeting diagnostic criteria for a major depressive episode or major depressive disorder.” (page 12)

**13: Results: Line 199-202: Please provide the p values in addition to the 95% CIs for the unadjusted and adjusted associations between ACEs and depression severity scores.**

RESPONSE: We now include the p-values in addition to the 95% CIs for the unadjusted and adjusted associations between cumulative number of ACEs and depression symptom severity scores (b=0.046; 95% CI, 0.038-0.053; p<0.001; adjusted b=0.050; 95% CI, 0.039-0.061; p<0.001).

**14: Results: Line 219-221: Please also provide p values in addition to 95% CIs for unadjusted and adjusted associations between major depressive disorder and ACEs number.**

RESPONSE: We now include the p-values in addition to the 95% CIs for the unadjusted and adjusted associations between cumulative number of ACEs and major depressive disorder (RR=1.184; 95% CI, 1.102-1.271; p<0.001; ARR=1.190; 95% CI, 1.109-1.276; p<0.001).

**15. Results: Line 221-224: Please provide p values in addition to 95% CIs for unadjusted and adjusted associations between suicidal ideation and cumulative ACE score.**

RESPONSE: We now include the p-values in addition the 95% CIs for the unadjusted and adjusted associations between cumulative number of ACEs and suicidal ideation (RR=1.164; 95% CI, 1.008-1.343; p=0.038; ARR=1.146; 95% CI, 1.001-1.311; p<0.048).

**16. Results Lines 225-239: for the linear regression models with categorical ACE score, the comparisons between major depressive disorder between ACE categories, and the association between ACE category and suicidal ideation, please provide p values for adjusted and unadjusted results. Please present the unadjusted results (in a table) and reference the location in the text here.**

RESPONSE: For the linear regression models with categorical ACE score as the explanatory variable and major depressive disorder and suicidal ideation as the outcome variables, we now include the p-values for the unadjusted and adjusted results. We also include the unadjusted results in a Supporting Table (S3 Table, referenced in the Results).

**17: Results: Lines 244-255: Please include p values together with 95% CIs for the associations between each of the types of ACE and depression symptoms, and please include a table of the unadjusted analyses.**

RESPONSE: The unadjusted analyses are now included in a Supporting Table (S4 Table) and referenced in the Results. We have also now included the p-values along with the 95% CIs for the ACEs with the strongest associations with depression symptom severity.

“In the adjusted models, associations were strongest for attempted or enacted sexual abuse (b=0.193; 95% CI, 0.127-0.259; p<0.001), observing violence toward the mother or grandmother (b=0.131; 95% CI, 0.088-0.174; p<0.001), and food and/or water insecurity (b=0.187; 95% CI, 0.137-0.238; p<0.001).” (page 23)

**18. Results: Lines 252-255: Please present the results with 95% CI and p value for the association between having a parent/adult in prison and suicidal ideation, as this is called out in the text.**

RESPONSE: Since we updated the scoring method for the ACE-IQ, two categories of ACE are now statistically significantly associated with suicidal ideation: living with an adult who was sent to prison or jail (ARR=2.654; 95% CI, 1.646-4.278; p<0.001) and food and/or water insecurity (ARR=1.882; 95% CI, 1.155-3.065; p=0.011). This is now noted in the text (page 23), along with the ARRs, 95% CIs, and p-values.

**19. Discussion: Please slightly reorganize the Discussion as follows: a short, clear summary of the article's findings; what the study adds to existing research and where and why the results may differ from previous research; strengths and limitations of the study; implications and next steps for research, clinical practice, and/or public policy; one-paragraph conclusion.**

RESPONSE: We have reorganized the Discussion in line with this comment. Additionally, in response to Reviewer #3’s comments, we moved some of the contextual information from the Discussion into the Introduction and Methods sections.

**20. Discussion: In the conclusion paragraph, please temper the statements to avoid inferring the direction of the relationship (ACE leads to depression) and policy/health systems recommendations based on this- while this is an interpretation of the findings, given the limitations mentioned and the cross sectional nature of the study, causal implications should be avoided (lines 381-383).**

RESPONSE: Thank you for this recommendation. In the first sentence of the Conclusion, we added the word “potential” to temper our statement – “Given the potential relationship between ACEs and adult depression…”.

We broke the next sentence (previously lines 381-383) into two sentences to further avoid inferring the direction of the relationship. This now reads: “By addressing multi-level factors contributing to these experiences, programs may reduce ACEs among children and adolescents. Based on our interpretation of the present findings, such intervention may prevent trajectories toward poor mental health during adulthood.” (pages 27-28)

**21. Table 1 and Table 2: Please remove the % sign from the columns headed “%” and please define all abbreviations such as ACE and HSCLD, in the legends.**

RESPONSE: We removed the % signs and added legends with abbreviations in Tables 1 and 2.

**22. Table 3 and Table 4: Please provide the actual p values associated with the adjusted results (rather than noting p<0.05, etc). In the legend, please provide the abbreviations for ACE, and HSCLD-15, and note which variables were adjusted for in these analyses. Please provide the unadjusted results (and reference in the text around Line 201, 220 and 223)- if desired this can be presented as a supporting information file. Please also clarify whether the suicidal ideation column is a comparison with total number of ACE, or cumulative ACE score (as mentioned in the text).**

RESPONSE: In Tables 3 and 4 we now include the p-values associated with the adjusted results (rather than noting statistically significant results with an asterisk). In the legend of each table, we also provide the abbreviations for ACE and HSCLD-15, and we note the variables that were adjusted for in the analyses. We have also included the unadjusted results as Supporting Tables (S1 Table; S3 Table).

We now refer to “cumulative ACE score” or “cumulative number of ACEs” – these terms both refer to the total number of ACEs that the participant experienced before age 18, out of a maximum of nine. We had previously used the term, “total number of ACEs”, to describe the same thing. However, we have now omitted this term from the text for consistency.

**23. S1 Appendix and S2 Appendix: Please note the actual p values, rather than * for p<0.05, for example. Please note in the legend of S1 Appendix what variables were adjusted for. For both, please also provide the unadjusted results.**

RESEPONSE: All of the Supporting Tables now include the p-values associated with the unadjusted and adjusted results (rather than noting statistically significant results with an asterisk). In the legends of the Supporting Tables reporting adjusted results, we include the variables adjusted for in the legends.

All of the unadjusted results are now reported in the Supporting Tables.

**24. Checklist: Please ensure that the study is reported according to the STROBE guideline and include the completed STROBE checklist as Supporting Information. When completing the checklist, please use section and paragraph numbers, rather than page numbers. Please add the following statement, or similar, to the Methods: "This study is reported as per the Strengthening the Reporting of Observational Studies in Epidemiology (STROBE) guideline (S1 Checklist)."**

RESOPNSE: The STROBE Checklist is now included as S1 Checklist. All items have been completed.

**Comments from the reviewers:**

**Reviewer #1:**

**This paper describes the association between reported childhood adversity and adult depression in rural Uganda.**

**I was asked for a statistical report and I interpret that to include all aspects of the design and conduct of the study.**

**Points of detail**

**1. Page 5 I am not sure what an emancipated minor is. Presumably not freed from slavery. Does it mean living without a parent or legal guardian?**

RESPONSE: On page 10, we now include a definition for emancipated minors:

“Emancipated minors were defined as persons younger than 18 years who are married, are pregnant, live with a biological child in the household, or are responsible for their own livelihood.”

As noted near the start of the Results section, only one 17-year-old emancipated minor was included in the study sample.

**2. Page 6 It seems surprising not to ask about abuse outside the household. Large-scale abuse of children by clerics and sporting coaches has been revealed in the media both in my country and that of the authors and elsewhere. Perhaps I am unduly cynical but is it really just a phenomenon of rich countries?**

RESPONSE: We thank the reviewer for this important point, and it is one that we have identified in our limitations section. We also include this as a limitation in the Methods and Findings section of the Abstract:

“The limitations of the study include the reliance on retrospective self-report as well as the focus on ACEs that occurred within the household.” (page 3)

**3. Page 6 The women do not seem to have been asked if they were cut. Perhaps it is either so common or so rare in Uganda that associations would not have been revealed.**

RESPONSE: While female genital mutilation (FGM) is still prevalent in some regions of the world, the practice has decreased in East Africa over the last several decades. Within East Africa, Uganda has one of the lowest prevalences of FGM, with the practice banned by the national government in 2010. The current estimated prevalence among 15 to 49-year-old women is 0.3%. The six districts with a higher prevalence of FGM are concentrated in the east. Across all regions, the prevalence is lowest in southwest Uganda, where the present study took place. Since FGM is so rare in southwest Uganda, participants were not asked if they had been cut.

Reference:

Female Genital Mutilation: Evidence from Uganda. Uganda Bureau of Statistics and UNICEF; 2020.

**4. Page 7 I think the authors probably used five categories rather than the four quintiles although that would have been possible. This leads to possible ambiguity. If fifths is not acceptable quintile categories is also used.**

RESPONSE: The procedures described by Filmer & Pritchett (ref #67 in the manuscript) produce a household asset wealth index on the continuous scale that ranged, in our study, from -2.788 to +19.10. Following Filmer & Pritchett, we then used these values to partition the sample into quintiles of household asset wealth such that the 326 people in the poorest quintile had asset index values ranging from -2.788 to -1.346, the 324 people in the 2^nd^ poorest quintile had asset index values ranging from -1.344 to -0.832, the 324 people in the 3^rd^ (middle) quintile had asset index values ranging from -0.832 to -0.198, the 326 people in the 4^th^ (less poor) quintile had asset index values ranging from -0.198 to +0.843, and the 326 people in the 5^th^ (least poor) quintile had asset index values ranging from +0.856 to +19.10. In the regression models, we designated the 1^st^ quintile (poorest) as the referent.

Reference:

Filmer D, Pritchett L. The effect of household wealth on educational attainment: Evidence from 35 countries. Population and Development Review. 2004;25(1).

**5. Page 8 A preliminary test for interaction would seem indicated before stratifying by age group. But why stratify? Categorising an essentially continuous variable wastes information (Altman and Royston, 2006; Royston et al., 2006) and leads to models which are often implausible as they predict the effect remaining flat within categories and then jumping to a new value at the category boundary.**

RESPONSE: We now include the preliminary test for interaction (using a product term between number of ACEs and age as a continuous variable) as the first column in S2 Table and provide the data from this test in the Results:

“In a multivariable linear regression model containing a product term between the cumulative number of ACEs and age, specified as a continuous variable, the estimated regression coefficient on the product term was statistically significant and negative, suggesting an interaction (b= -0.001; 95% CI, -0.0014- -0.0006; p<0.001): the estimated association between ACEs and depression symptom severity weakened with age (S2 Table). Consistent with this regression model, when we fit regression models stratified by age bin, the estimated association between the cumulative number of ACEs and depression symptom severity was largest among younger adults (b=0.060; 95% CI, 0.047-0.073; p<0.001) and adults (b=0.064; 95% CI, 0.056-0.071; p<0.001) and weakest among older adults (b=0.031; 95% CI, 0.017-0.046; p=0.001).” (pages 18-19)

Additionally, we provide details in the Data Analysis section of the Methods to explain this preliminary test for interaction: “To determine the extent to which the associations remained statistically significant across the age range, we first fitted a multivariable linear regression model containing a product term between the cumulative number of ACEs and age, specified as a continuous variable. We then conducted further analyses stratified by age…” (page 13).

**6. Page 12, Table 2 There are nine categories here not the 16 mentioned on page 6. I think I may be able to guess what is going on especially after looking at S2 but perhaps the authors might make it explicit here. If I am wrong and some have been dropped then that would seem sub-optimal.**

RESPONSE: The modified ACE-IQ, which is now included as a supporting information file (S1 Appendix), has 16 questions. These questions fall into nine types of ACE, as summarized in Table 2. In our initial analyses, we had calculated the total ACE score using all 16 questions. We have since modified our analysis plan in response to the reviewer feedback. Rather than using all 16 questions in the total ACE score, we recalculated the total ACE score using the nine types of ACE. This is described on page 11:

“The 16 items on the ACE-IQ were then grouped into nine types of ACE: 1) physical abuse; 2) verbal or emotional abuse; 3) attempted or enacted sexual abuse; 4) residence with an adult who used alcohol or drugs; 5) residence with an adult who had mental illness or who attempted suicide; 6) parents separated or divorced; 7) residence with an adult who was sent to prison or jail; 8) observed violence toward mother or grandmother; and 9) food and/or water insecurity. These nine binary variables were summed to calculate the cumulative number of ACEs (S2 Appendix).”

We have re-fitted all analyses in the manuscript and supporting information files to reflect the change in our scoring method.

**7. Page 13 I would not have said that the difference between 0.042, 0.043 and 0.022 were minor as the last is about half the other two.**

RESPONSE: We agree with the reviewer that the difference in the strength of the association between cumulative ACE score and depression symptom severity between adults and older adults in our prior models was not minor, as we had incorrectly stated in the text.

We have now updated all analyses in the manuscript using the updated scoring method for the ACE-IQ (maximum cumulative score of 9 to reflect the 9 types of ACE rather than the 16 questions on the ACE-IQ). In the updated models (S2 Table), the adjusted beta coefficient equals 0.060 for younger adults, 0.064 for adults, and 0.031 for older adults. We have updated the text in the manuscript, as reflected in response to point 5, above.

**8. Page 18 The prevalences here do not seem very close to those in Table 2. The authors mention as a possible limitation not being able to ensure confidentiality of the interviews but did reduce the prevalence of all the ACE relative to epidemiological studies?**

RESPONSE: The prevalence of sexual violence and physical abuse among boys and girls in Uganda (previously reported on page 18) has now been moved to the Methods section (page 9), prior to our report of the prevalence of probable depression among adults.

Multiple factors may account for the difference in prevalences of ACEs reported in the ‘Violence Against Children Survey’ vs. those reported in our study. First, the ‘Violence Against Children Survey’ surveyed adolescents and young adults about their recent experiences rather than eliciting retrospective reports from adults. Recall of events experienced during childhood may decrease over time. Additionally, the nationally-representative survey asked about any experiences of violence, perpetrated in the home or in the community. As previously noted, the modified ACE-IQ primarily asked participants about events that occurred within the household. In the ‘Violence Against Children Survey’, only 4.5% of girls who experienced sexual abuse prior to age 18 reported that the first incident was perpetrated by a family member. Although items 5 and 6 on the modified ACE-IQ do not specify whether or not the attempted or enacted sexual abuse occurred in the household, both questions first mention a parent. Furthermore, in the context of the broader instrument, which largely focuses on events within the household, this condition may have been assumed by participants. Last, the ‘Violence Against Children Survey’ included a sample of adolescents and young adults across the entire country. The authors identified some regional differences in violence against children; however, they were unable to distinguish between experiences of children in rural vs. urban settings. Some preliminary evidence from the survey suggests that females in more urbanized areas may be more likely to have had sex in exchange for material support. There may be other national differences across regions as well as in rural vs. urban settings that account for the lower prevalences in our study.

**Point of more substance**

**In pages 19 and 20 the authors present their recommendations for the health service in Uganda. It seems to me that their research design has not justified this as it has not ruled out reverse causation. Presumably many of the people currently depressed may have had mental health problems before age 18 although not necessarily depression. That may have influenced the way people behaved towards them leading to at least some of the items on the ACE schedule becoming more prevalent or it may be that their childhood problems modified their perception of the world and events in it. In either of those cases the recommendation would presumably be to increase funding for child and adolescent mental health services rather than reducing adverse events.**

RESPONSE: In response to this point, we have now modified part of our Discussion to address implications for any potential reverse causality, as described in your comment:

“Due to the cross-sectional nature of this study, we were unable to determine causality between ACEs and poor mental health during adulthood. For example, adults may react differently to children who have mental health problems, including both internalizing and externalizing disorders, thereby predisposing these children and adolescents to certain types of ACEs. Furthermore, early mental health problems may modify individuals’ perceptions of the world and their experiences. As we are not able to exclude reverse causality as a potential explanation for the observed associations, the present findings may have implications for mental health care among both children and adults.” (page 27)

**Summary - No major issues. Michael Dewey**

**References**

**D G Altman and P Royston. The cost of dichotomising continuous variables. *British Medical Journal*, 332:1080, 2006.**

**P Royston, D G Altman, and W Sauerbrei. Dichotomizing continuous predictors in multiple regression: a bad idea. *Statistics in Medicine*, 25:127-141, 2006.**

**Reviewer #2:**

**This is a very good paper, thorough and well constructed. It follows closely the established ACE methodology conducted by the CDC in the US, and replicated in other high-income settings. Its findings are perhaps not totally surprising, but they are nonetheless very important. We have always assumed that ACE's work in similar ways in LMIC, but this is a very robust investigation of what is actually an empirical question, and the findings are very clear. In fact, they are so close to the findings of the original CDC study (which had a very similar method - retrospective reporting of a population cohort) that this is worth remarking on. ACE impact people in Sub-Saharan Africa just like they do in America. This does matter and should be published.**

**I would have been interested to see an analysis by gender, but I don't think this should be required at all - just a thought (and gender is in the models). I should add that I'm not so familiar with the adult mental health literature in Eastern Africa, so I have taken as accurate that this kind of study has not been conducted before in the region.**

**I would recommend this paper for publication, and commend the authors. I'd be interested to know what the objections of previous reviewers were. I would also be very interested for future papers to see the impacts of ACEs on other health, economic and social outcomes. Lucie Cluver.**

RESPONSE: We thank the reviewer for the positive feedback on our manuscript. We agree that further investigations into the impacts of ACEs on other outcomes are warranted. Members of our research team are currently working on analyses that look at associations between ACEs and alcohol use and cardiometabolic indicators during adulthood. Additionally, our team is working on an analysis that investigates associations between parents’ reported ACEs and their children’s mental health.

**Reviewer #3:**

**Overall, the methodology and writing is solid. To make an impact, it needs to go further: it needs to really contextualize this piece of research in the larger field, it needs to make a case for what new information it is bringing to the field, and it needs to dive deeper into a rich discussion of what those findings mean for practice. As written, the discussion is not sufficiently developed.**

RESPONSE: We thank the reviewer for raising these important considerations. We hope that our changes throughout the manuscript, detailed below, strengthen its impact and provide important implications for practice.

**1. The literature review seems to neglect a lot of work that has been done on ACEs and psychological health in Africa. For example, Cluver et al in South Africa; Manyema et al with young adults in South Africa, and Kidman et al in Malawi have all explicitly studied ACEs and suicidality, depression or a similar outcomes. These are not perfect parallels to the research question or age group here, but would provide additional context.**

RESPONSE: We thank the reviewer for pointing out these additional studies. We have edited the final paragraph of the Introduction to include evidence from other studies conducted in sub-Saharan Africa. Research by Cluver et al. (2015) and Kidman, Piccolo & Kohler (2020) demonstrate associations between adverse childhood experiences and depression and suicidality among adolescents and young adults. However, these studies do not assess the persistence of these associations across the age range. Additionally, these and other studies from the region do not assess for major depressive disorder. Our study fills these gaps.

“Similarly, a prospective study among South African adolescents found a statistically significant graded relationship between the cumulative number of ACEs and suicide behavior after adjusting for baseline suicidality (Cluver et al., 2015). Two studies of children and adolescents in Uganda found that depression was associated with loss of a parent and alcohol consumption and domestic violence. While these and other studies have indicated a high prevalence of ACEs and associations with depression and suicidality among children, adolescents, and young adults in sub-Saharan Africa (Kidman, Piccolo & Kohler, 2020; Manyema & Richter, 2019), to our knowledge, no study has yet estimated the associations between ACEs and major depressive disorder or suicidal ideation within a whole-population sample of adults in this setting.” (page 8)

References:

Cluver L, Orkin M, Boyes ME, Sherr L. Child and adolescent suicide attempts, suicidal behavior, and adverse childhood experiences in South Africa: A prospective study. Journal of Adolescent Health. 2015;57(1):52-9.

Kidman R, Piccolo LR, Kohler HP. Adverse childhood experiences: Prevalence and association with adolescent health in Malawi. American Journal of Preventive Medicine. 2020;58(2):285-93.

Manyema M, Richter LM. Adverse childhood experiences: prevalence and associated factors among South African young adults. Heliyon. 2019;5(12):e03003.

**2. The introduction states that this line of research has been done in HIC, but not LMIC. However, there is no nuanced discussion of how these contexts might matter to the relationship under study.**

RESPONSE: We have moved information that was previously included in the Discussion into the Introduction. This provides context on how poverty in LMICs may compound the pathway between ACEs and adult depression.

“Childhood adversity is prevalent across LMICs, with evidence demonstrating the exacerbating effects of poverty and family violence on other childhood traumas. Negative socioeconomic conditions in general, including insecure access to food and water, present additional obstacles to healthy development, with documented negative effects on school attendance, health, and wellbeing. Prior research from other contexts also indicate that neighborhood poverty is positively associated with abuse, child maltreatment, and household dysfunction including high caretaker stress and depression. Living in unsafe, “stressogenic” environments characterized by economic precarity, a lack of resources, and violence increases children and adolescents’ exposure to stress and trauma. Furthermore, as “poverty begets trauma” and “stress begets stress”, individuals who grow up in such environments are at a higher risk of experiencing psychopathology as adults. Thus, the pervasive poverty in some LMICs further underscores the importance of studying the relationship between ACEs and adult mental ill health.” (page 7)

**3. There is also little on how ACEs get embodied. A sentence or two on how ACEs translate into later depression would be helpful.**

RESPONSE: We had previously included context on how ACEs get embodied and are translated into later depression in the Discussion. As the reviewer notes, this information did not belong in the Discussion section. We have now moved this text to the Introduction.

“Research has found that ACEs increase children and adolescents’ risk of having low resilience factors. The magnitude of this association may partially depend on individual-level vulnerability. A study from Australia directly testing the diathesis-stress model for depression found that adults who had high pre-dispositional vulnerability and who had experienced more stressful, adverse life experiences were at the highest risk of developing depression.” (page 6)

After noting that the effects of ACEs on adult mental health may be cumulative, we include information on the cumulative disadvantage theory:

“The cumulative disadvantage theory posits that early advantage and disadvantage, including that which results from genetic and environmental factors, may compound, resulting in markedly differing trajectories over time.” (page 7)

**4. Why was this particular area (Nyakabare Parish) chosen for this study? Why a census instead of a random sample of a larger area?**

RESPONSE: The parent study was principally designed as a sociocentric social network study, which requires data on all individuals in the population. We have added additional details to the manuscript describing how Nyakabare Parish was chosen as the study site:

“Through an iterative process involving field site visits and informal conversations with local leaders and other prominent village residents, we selected Nyakabare Parish as the study site. It was smaller than many other parishes in the region, which facilitated our team’s ability to capture a whole-population sample; the local leaders were supportive of engaging area residents in a population-based household survey; and the local leaders stated that there was relatively less non-governmental organization involvement in the area, in terms of service delivery or other development activities.” (page 9)

**5. The paper states that the ACE-IQ was modified, but it isn't clear how it was modified. Please provide details. How was it coded? It mentions a point for each item, but typically the coding for the ACE-IQ is a bit more complicated and based at least on a point per type of adversity (which can cover multiple items). Table 2 seems to list these, indicating the range should be 0-9. Is this correct? Later you refer to 16 - but that should be questions not ACEs. And why was food insecurity included? That decision is fine, but a justification is needed. I think some of the other questions are a little different too. This will be confusing when researchers try to compare ACE prevalence across populations/papers without adequate descriptions.**

RESPONSE: The modification of the ACE-IQ is now described in the Methods:

“We modified the ACE-IQ following an iterative process of focus group discussion with key informants. Some of the original ACE-IQ items were dropped because they were either poorly understood or thought to be less applicable for our population. Additional items on food and water insecurity were added because both food and water insecurity are common in this rural setting.” (page 10-11)

The modified ACE-IQ is now included as a supporting information file (S1 Appendix) and had 16 questions. Response options were similar to the original ACE-IQ (e.g., “never”, “once”, “a few times”, “frequently”). The 16 questions are clustered into nine categories of ACE, as summarized in Table 2. In our initial analyses, we had calculated the total ACE score using all 16 questions, coded as binary variables. In response to the reviewer’s feedback, we have since modified our analysis plan. Rather than using all 16 questions in the total ACE score, we recalculated the total ACE score using the nine types of ACE, as indicated by the WHO’s ACE-IQ binary version for scoring (ACE-IQ manual, page D1.1, Table 1). For example, if a participant experienced any physical abuse, as defined by item 3 and/or item 4 on the modified ACE-IQ, they received a ‘1’ for physical abuse. If the participant reported never experiencing physical abuse on items 3 or 4, they received a ‘0’ for physical abuse.

“The 16 items on the ACE-IQ were then grouped into nine types of ACEs: 1) physical abuse; 2) verbal or emotional abuse; 3) attempted or enacted sexual abuse; 4) residence with an adult who used alcohol or drugs; 5) residence with an adult who had mental illness or who attempted suicide; 6) parents separated or divorced; 7) residence with an adult who was sent to prison or jail; 8) observed violence toward mother or grandmother; and 9) food and/or water insecurity. These nine binary variables were summed to calculate the cumulative number of ACEs (S2 Appendix).” (page 11)

We have re-fitted all analyses in the manuscript and supporting information files to reflect the change in our scoring method.

Reference:

World Health Organization. Adverse Childhood Experiences International Quesionnaire (ACE-IQ). Geneva, Switzerland: World Health Organization. 2012. Available at: https://www.who.int/violence_injury_prevention/violence/activities/adverse_childhood_experiences/en/. Accessed December 10, 2020.

**6. It would be helpful to have a bit more on the algorithm for the depression diagnosis in the paper, especially since it is the key outcome.**

RESPONSE: We have added an in-depth description of our algorithm for the major depression diagnosis:

“The original HSCLD-15 includes 15 items. Local modifications involved dropping one item (“feeling trapped”) and adding one item (“don’t care what happens to your health”). These 15 items were then categorized into the nine DSM ‘A’ criteria: A1) depressed mood, A2) diminished interest or pleasure, A3) significant weight loss or change in appetite, A4) insomnia or hypersomnia, A5) psychomotor agitation, A6) fatigue or loss of energy, A7) feeling worthless or guilty, A8) diminished ability to think or concentrate; and A9) recurrent thoughts of death. Participants who experienced an HSCL-D item ‘much’ or ‘very much’ in the past week were classified as meeting the respective DSM ‘A’ criteria. We then summed the total number of criteria met, out of a maximum of nine. If a participant reported at least 5 criteria, *and* met either the A1 criterion (depressed mood) *or* the A2 criterion (diminished interest or pleasure), they were identified as likely meeting diagnostic criteria for a major depressive episode or major depressive disorder.” (page 12)

**7. Analyses adjust for clustering at the village level, but what about the household level? If you only interviewed one per household, this should be in the methods. However, based on the numbers presented, I don't think this is the case.**

RESPONSE: We clustered standard errors at the village level, following general econometric guidance that, in the setting of multiple levels of clustering, consistent confidence intervals will be obtained by using cluster-robust variance estimated at the highest level of clustering (Angrist & Pischke, 2009; Cameron & Miller, 2015).

Current guidance notwithstanding, our estimates are robust to clustering a lower level of analysis. In the table at the end of this document, we replicate the estimates from Table 3 using standard errors clustered at the household level. As can be observed in the table:

- For the depression symptom severity regression model, the confidence interval widens (i.e., the t-statistic decreases). In the linear regression model clustered at the village level, t=11.15. In the model clustered at the household level, t=10.87. Since the number of clusters is small, as in our case, with only 8 villages, we also did a wild bootstrap test (Cameron & Miller, 2015). In the wild bootstrap test, t=11.15.
- For the suicidal ideation model, the confidence internal widens (i.e., z-score decreases), and the estimated relative risk is not statistically significant.
- For the major depressive disorder model, the confidence interval narrows (i.e., z-score increases).

Following general econometric guidance, we have elected to retain the Table 3 estimates as presented in the manuscript but provide the alternative estimates here for the benefit of the reviewer. We are open to revisiting this issue if deemed necessary by the statistical editor.

References:

Angrist & Pischke, *Mostly Harmless Econometrics*, 2009

Cameron AC, Miller DL, A practitioner’s guide to cluster-robust inference, J Hum Resources. 2015;50:317-372

**8. Do you have the statistical power to examine individual ACEs and suicide ideation?**

RESPONSE: We did not have statistical power to examine associations between individual ACEs and suicidal ideation. As described in Table 1, the number of suicidal ideation events was N=28. This is smaller than the number of major depressive disorder events (N=159) by a factor of five. Given the limited statistical power, the analyses for the individual ACEs are presented in the appendix as secondary analyses rather than in the main text.

**9. What value do you get out of the individual ACE models? Given the similarity in coefficients, are they important alone, or do they serve as a proxy for overall ACE exposure? For these analyses to be included, it would be essential to motivate them the introduction. Why isn't a cumulative score a better indicator; why would individual ACEs matter and which ones would you expect to see generate a large association? In the absence of such, this comes off as rather exploratory.**

RESPONSE: The individual ACE models were included as secondary analyses to determine if individual types of ACE have differential impacts on mental health. As noted in the Methods on page 14, these models allowed “us to estimate the associations between each type of ACE and depression symptom severity, major depressive disorder, and suicidal ideation, without assuming that the different ACE had equivalent associations.”

In the Introduction, we describe research that have indicated differing associations between specific ACEs and adult mental health:

“In addition to the cumulative nature of ACEs, some experiences may be more salient than others; one study, for example, found stronger associations between negative mental health outcomes and child maltreatment than with household dysfunction (Negriff, 2020). An umbrella review of 19 meta-analyses found strong associations between childhood sexual abuse and depression in adulthood, as well as other psychosocial and psychiatric outcomes (Hailes et al, 2019).” (page 7)

The studies we cite from Uganda and South Africa estimated associations between specific types of ACE (e.g., loss of a parent, alcohol consumption, emotional neglect, sexual abuse) and depression. Our study extends prior research to assess if these associations hold up across the age range and for other types of ACE.

If different ACEs have differential impacts on adult depression and suicidal ideation in this setting, recommendations for policy and practice should target these ACEs.

References:

Negriff S. ACEs are not equal: Examining the relative impact of household dysfunction versus childhood maltreatment on mental health in adolescence. Social Science and Medicine. 2020;245:112696.

Hailes HP, Yu R, Danese A, Fazel S. Long-term outcomes of childhood sexual abuse: An umbrella review. Lancet Psychiatry. 2019;6(10):830-9.

**10. The paragraph starting with line 275 seems to fit better in an introduction. I am not clear how it sheds further light on the new findings. I am also not clear where poverty comes into the discussion of ACEs and depression.**

RESPONSE: The majority of this paragraph has now been moved to the Introduction to contextualize how Uganda and other LMICs may differ from HICs in the embodiment of ACEs into adult mental health problems. The prevalence data on violence against children in Uganda has been moved to the *Study setting and population* subsection of the Methods (page 9).

**11. The last sentence of that paragraph implies that the study found little access to support or resources; this isn't reported in the paper.**

RESPONSE: This sentence has been removed and we have moved information on limited mental health support in Uganda to the *Study setting and population* subsection of the Methods (page 9).

**12. The paragraph starting with line 289 also seems out of place. This is background material, and better suited for an introduction. It is not again related to the findings. The focus on "stressogenic" environments and poverty in particular seems to be introduced for the first time here, and is background - it isn't something we learn from or sheds light on the findings.**

RESPONSE: As described above, this paragraph has now been moved to the introduction to provide additional context on how ACEs are embodied and may translate into later depression (page 7).

**13. The discussion on implications seems like it could have been written before this study was conducted. It doesn't answer new questions about what should be done using your new findings. The authors start to bring in some of this - such as the fact that ACEs were incredibly high compared to other contexts - but don't drill down. They also start to acknowledge the limited mental health resources available in Uganda, but don't really go into how the new information on ACEs relates to this. The authors make the argument that the high rates of depression warrant more investment in mental health, but this isn't the core of the paper/findings - in fact, these rates were given as background. What other approaches could be useful in this context, given the new info? If there are Ugandan authors, perhaps they can help.**

RESPONSE: As suggested by Reviewer #1, our study design does not allow us to rule out reverse causality. We have added a description of how this may have implications for improved mental health systems for both children and adults in the Discussion:

“Due to the cross-sectional nature of this study, we were unable to determine causality between ACEs and poor mental health during adulthood. For example, adults may react differently to children who have mental health problems during childhood and/or adolescence, including both internalizing and externalizing disorders, thereby predisposing these children and adolescents to certain types of ACEs. Furthermore, early mental health problems may modify individuals’ perceptions of the world and their experiences. As we are not able to exclude reverse causality as a potential explanation for the observed associations, the present findings may have implications for mental health care among both children and adults.” (page 27)

Table 3 replicated, using standard errors clustered at the household level rather than the village level. Adjusted linear and Poisson regression models estimating associations between number of ACEs and depression symptom severity, major depressive disorder, and suicidal ideation.

|  | **Depression Symptom Severity** | | **Major Depressive Disorder** | | **Suicidal Ideation** | |
| --- | --- | --- | --- | --- | --- | --- |
|  | **Adjusted b (95% CI)** | ***p*-value** | **Adjusted RR (95% CI)** | ***p*-value** | **Adjusted RR (95% CI)** | ***p*-value** |
| **Cumulative No. ACEs** | 0.050 (0.041-0.059) | <0.001 | 1.190 (1.114-1.270) | <0.001 | 1.146 (0.963-1.364) | 0.126 |
| **Female** | 0.178 (0.141-0.215) | <0.001 | 1.858 (1.338-2.580) | <0.001 | 2.620 (1.065-6.444) | 0.036 |
| **Age (years)** | 0.003 (0.001-0.004) | <0.001 | 1.002 (0.994-1.011) | 0.586 | 0.989 (0.963-1.015) | 0.395 |
| **Completed Primary School** | -0.087 (-0.137-0.036) | 0.001 | 0.471 (0.333-0.667) | <0.001 | 0.365 (0.149-0.896) | 0.028 |
| **Married** | -0.068 (-0.109- -0.028) | 0.001 | 0.712 (0.528-0.960) | 0.026 | 1.178 (0.536-2.592) | 0.683 |
| **HIV Positive** | -0.035 (-0.098-0.027) | 0.268 | 0.809 (0.520-1.261) | 0.350 | 1.071 (0.380-3.021) | 0.896 |
| **Wealth Quintile** |  |  |  |  |  |  |
| Poorest |  |  |  |  |  |  |
| 2nd | -0.060 (-0.124-0.004) | 0.066 | 0.783 (0.517-1.186) | 0.248 | 0.311 (0.086-1.130) | 0.076 |
| 3rd | -0.040 (-0.104-0.023) | 0.215 | 0.804 (0.518-1.246) | 0.329 | 0.856 (0.328-2.238) | 0.752 |
| 4th | -0.068 (-0.132- -0.003) | 0.040 | 0.644 (0.392-1.059) | 0.083 | 0.284 (0.065-1.242) | 0.094 |
| Richest | -0.035 (-0.105-0.035) | 0.330 | 1.008 (0.630-1.614) | 0.972 | 1.019 (0.347-2.991) | 0.973 |
| **Constant** | 1.246 (1.161-1.330) | <0.001 | 0.065 (0.034-0.122) | <0.001 | 0.018 (0.004-0.090) | <0.001 |
| **Observations** | 1,602 | | 1,602 | | 1,602 | |
| **R^2^ and Pseudo R^2^** | 0.157 | | 0.076 | | 0.080 | |
| Abbreviations: b, beta coefficient; RR, relative risk; CI, confidence interval; ACEs, adverse childhood experiences | | | | | | |
