# Supplementary material for: Adverse childhood experiences, adult depression, and suicidal ideation in rural Uganda: A cross-sectional, population-based study
Source: PLoS Med. 2021 May 12;18(5):e1003642. doi: 10.1371/journal.pmed.1003642 (PMC8153443; doi:10.1371/journal.pmed.1003642)
Supplement: Attachment — Submitted filename: acesdepression_response-to-reviewers-2_plosmed_20210423.docx [file pmed.1003642.s012.docx]

**Requests from Editors:**

**1. From the academic editor: Methods: Line 133-134: Is it relevant to say that the study is part of a population-based cohort? Please clarify to avoid confusion.**

RESPONSE: We have removed the word “cohort” from this sentence to avoid confusion.

**2. From the academic editor: Methods: Line 200-201: Although the Hopkins Symptom Checklist has been validated in Uganda, please confirm whether it has been validated in this Ugandan language or cultural group (or in a rural setting).**

RESPONSE: The Hopkins Symptom Checklist has been modified and validated for use in Nyakabare Parish, the setting in which this study took place (Ashaba et al 2018).

We have revised the sentence for clarity and moved the modifications made to the instrument to this section: “The self-report instrument assesses for symptoms of depression over the past week and has been modified and validated for use among Runyankore-speaking populations [59,60]. The original HSCL-D includes 15 items. Local modifications involved dropping one item (“feeling trapped”) and adding one item (“don’t care what happens to your health”) [42,60].”

References:

Ashaba S, Kakuhikire B, Vorechovska D, et al. Reliability, validity, and factor structure of the Hopkins Symptom Checklist-25: Population-based study of persons living with HIV in rural Uganda. AIDS Behav 2018;22(5):1467-74.

**3. We suggest revising the title to: “Adverse childhood experiences, adult depression and suicidal ideation in rural Uganda: A cross-sectional, population-based study”**

RESPONSE: We have revised the title to “Adverse childhood experiences, adult depression, and suicidal ideation in rural Uganda: A cross-sectional, population-based study”.

**4. Please revise the short title to: “Adverse childhood experiences and adult depression in Uganda”**

RESPONSE: We have revised the short title accordingly.

**5. Data availability statement: Please update the statement at this time, with your plan for making the anonymized data and code available (with links for access to anonymized data and Stata code in a repository or similar).**

RESPONSE: Over the next few days, we will upload the anonymized data and code to GitHub. On the cover page, we have added the following statement:

“Data Availability: All files are available from the following GitHub repository: <https://github.com/esatinsky/acesdepression_paper>”

**6. Abstract: Methods and Findings: Line 10: Should “elicited” be “assessed” or similar? (“...ACES were elicited using a modified…”)**

RESPONSE: We have replaced the word “elicited” with “assessed”.

**7. Abstract: Line 15: Please remove “significant” before suicidal ideation.**

RESPONSE: We have removed the word “significant” from this line.

**8. Introduction: Line 122 (and throughout text): For in-text references, where a range or list of references are noted within brackets, please do not include spaces [31,32].**

RESPONSE: We have updated the citations so that any range or list of references is noted within brackets and separated by a comma without spaces.

**9. Introduction: Line 122: Please qualify “...no study has yet…” with “to our knowledge” or similar.**

RESPONSE: We have revised these sentences to exclude the priority claim altogether: “These and other studies have indicated a high prevalence of ACEs and associations with depression and suicidality among children, adolescents, and young adults in sub-Saharan Africa [31,32,36,37]. To address the gap in the literature on ACEs and mental health among adults, we aimed to estimate the associations between ACEs and depression symptom severity, major depressive disorder, and suicidal ideation in a population-based sample of adults in rural Uganda.”

**10. Methods: Line 159: Please provide the number of adults represented by the 758 households.**

RESPONSE: The initial population census included the enumeration of 1,933 adults across the 758 households. These individuals were potential participants for the parent study, prior to eligibility assessment. This line has been revised as follows, “Prior to starting the study, a population census was conducted, including the enumeration of all 1,933 adults across the 758 households in the parish.”

**11. Methods: Line 168: Please provide the number of eligible adults.**

RESPONSE: This sentence has been revised as follows, “Between 2016 and 2018, a team of research assistants visited the homes of all 1,795 eligible adults to request study participation and obtain written informed consent.”

Please note, we have revised the dates of data collection from 2017-2018 to 2016-2018, as data collection began during the last two weeks of December 2016. Additionally, we updated the first sentence of the Results to include the number of eligible adults (1,795) rather than the number of eligible adults approached for participation (1,725). These numbers are different since some individuals could not be found during the data collection period. This revision changed the response rate from 95% to 91%.

**12. Methods: Line 267-268: Please clarify the rationale for not adjusting at the household level, in line with the response to reviewer 3, comment 7. For example, in the response to reviewers it is mentioned that the estimates in Table 3 are consistent between clustering at village vs household level, but you also note that confidence intervals become less precise adjusting for household clustering. Please consider incorporating this into the supporting information as an additional analysis.**

RESPONSE: We have incorporated our response to reviewer 3, comment 7 into the manuscript. At the end of the Methods section, we now include rationale for why we adjusted for clustering at the village level:

“Following general econometric guidance [73,74], all regression models included adjustment for clustering at the village level. In the setting of multiple levels of clustering, consistent confidence intervals will be obtained by using cluster-robust variance estimated at the highest level of clustering. In a sensitivity analysis, we re-fitted the primary regression models adjusting for clustering at the household level.”

At the end of the first paragraph of the Analyses subsection of the Results, we refer to this sensitivity analysis: “In sensitivity analyses, the estimates remained qualitatively similar when we clustered the standard errors at the household level (S2 Table).”

**13. Results: Line 299: Please remove “significant” before suicidal ideation.**

RESPONSE: We have removed the word “significant” before “suicidal ideation”.

**14. Discussion: Thank you for including the sections describing the strengths and limitations of the work, as well as implications for future research, practice, and policy. If possible, please consider expanding on the paragraph between lines 380-386, where you describe your findings in the context of the existing literature.**

RESPONSE: We have expanded on this paragraph to bring in additional literature.

“The estimated associations between ACEs and major depressive disorder and suicidal ideation are reflective of research showing strong associations between high levels of adversity and major depressive disorder among older adults [75], as well as research indicating associations between ACEs and suicidal ideation and suicide attempts across the lifespan [13,76]. Additionally, the finding that participants who reported food and water insecurity during childhood were at higher risk for suicidal ideation is consistent with a previously published multi-country study showing an increased odds of suicide attempt among adolescents reporting severe food insecurity [77].”

References:

Kim Y, Kim K, Chartier KG, Wike TL, McDonald SE. Adverse childhood experience patterns, major depressive disorder, and substance use disorder in older adults. Aging Ment Health. 2021;25(3):484-91.

Thompson MP, Kingree JB, Lamis D. Associations of adverse childhood experiences and suicidal behaviors in adulthood in a U.S. nationally representative sample. Child Care Health Dev. 2019;45(1):121-8.

Konyangi A, Stubbs B, Oh H, Veronese N, Smith L, Haro JM, et al. Food insecurity (hunger) and suicide attempts among 179,771 adolescents attending school from 9 high-income, 31 middle-income, and 4 low-income countries: A cross-sectional study. J Affect Disord. 2019;248:91-8.

**15. Discussion: Line 377: Please consider changing the wording of “risk of developing depression” as the study is cross-sectional it may be more conservative to avoid implying any temporal nature of the association.**

RESPONSE: This sentence has been revised to avoid implication of a temporal association: “Furthermore, we estimated a graded association, whereby the prevalence of depression was highest among individuals who reported the highest category of ACEs (≥6 experiences).”

In the Results section, we have revised the language similarly: “The Poisson regression models estimating the association between categorical ACEs score and major depressive disorder also indicated a graded increase in the prevalence of depression across ACE categories.” (page 21)

**16. Discussion: Line 384-386: Please revise this sentence to make the meaning more clear: “Finally, we demonstrate that our findings are such that only strong confounding by unobserved variables could completely explain them.” This could be modified to “...results suggest that our findings are unlikely to be completely explained by unobserved variables, for example…” or similar.**

RESPONSE: This sentence has been simplified as suggested: “Finally, the results suggest that our findings are unlikely to be completely explained by confounding from unobserved variables.”

**17. Discussion: Line 472: Please consider tempering “prevent” to “improve” here, to avoid any causal implications.**

RESPONSE: We have replaced the word “prevent” with “improve”.

**18. References: Please use the "Vancouver" style for reference formatting, and see our website for other reference guidelines** [**https://secure-web.cisco.com/1kmqX8ao6pG8i6CPOx__TDoML8aTY7ew_CQ38eWihj78GBdpHkKWl3ug8BRRpimbc_Y0CCmyGa5MQlr7f2U3R26z8mFEwukATvZ1Mw7jKYt4YcQqC0LmQaJMn28zT434YXFTZ_shVGxqdODBXNx3h88fGBpEIEctLoXXsT_nthy4u4PWrW4f9CZuBKPFTTJXuhJfJA1Nmhg9N8KSdkFDTe4LcSUP4ykmPBdvnGfC2tSRFsEBYyFy6KWLdJOK_0DeKX9x55VnjnC_5fLRXFBWWJg/https%3A%2F%2Fjournals.plos.org%2Fplosmedicine%2Fs%2Fsubmission-guidelines%23loc-references**](https://secure-web.cisco.com/1kmqX8ao6pG8i6CPOx__TDoML8aTY7ew_CQ38eWihj78GBdpHkKWl3ug8BRRpimbc_Y0CCmyGa5MQlr7f2U3R26z8mFEwukATvZ1Mw7jKYt4YcQqC0LmQaJMn28zT434YXFTZ_shVGxqdODBXNx3h88fGBpEIEctLoXXsT_nthy4u4PWrW4f9CZuBKPFTTJXuhJfJA1Nmhg9N8KSdkFDTe4LcSUP4ykmPBdvnGfC2tSRFsEBYyFy6KWLdJOK_0DeKX9x55VnjnC_5fLRXFBWWJg/https%3A%2F%2Fjournals.plos.org%2Fplosmedicine%2Fs%2Fsubmission-guidelines%23loc-references)

**(For example, in Ref 4, PLoS Medicine should be PLoS Med).**

RESPONSE: The references have been updated to reflect the formatting guidelines.

**19. Supporting Information: Thank you for including the revised supporting information documents. Please submit a finalized version for each item (rather than the tracked changes versions). It would be helpful for each item to be included in an independent file and labeled as such (S1_Analysis Plan, S1_Checklist, S1_Table, etc.)**

RESPONSE: We have saved each supporting information document as an independent file.

**20. S3 Appendix (Analysis plan): It would be helpful to include in this Prespecified Analysis Plan document a brief mention of the specific outcomes and analyses planned, to go along with your statement that all the described analyses were specified at the outset of the study. Thank you for thoroughly documenting the changes made to the analyses during the course of peer review.**

RESPONSE: We have updated S3 Appendix with a brief description of the outcomes and analyses planned at the outset of the study. We also added to the end of the Appendix a brief description of the sensitivity analysis incorporated in response to reviewer feedback.

**Comments from Reviewers:**

**Reviewer #1: The authors have addressed my points.**

**I may not have been sufficiently clear about the quintiles issue. My point was that this is potentially ambiguous as there are only four quintiles. It would have been possible to use them in the regression but that is not what the authors did. I agree this is a bit picky. I suggested quintile categories or even the everyday English word fifths but if the authors insist I would not want to man the barricades over it. I realise I am fighting a losing battle here anyway and will soon have to accept people talking about dividing a sample into two medians.**

**Michael Dewey**

RESPONSE: We have revised “wealth quintiles” to “wealth quintile categories”.
